# Combination of rhinoshave and fractional ablative CO_2_ laser therapy for fine contouring of pronounced rhinophyma – A monocentric retrospective study with long‐term follow‐up

**DOI:** 10.1111/ddg.15692

**Published:** 2025-04-03

**Authors:** Conrad Hempel, Anna‐Theresa Seitz, Viktor Schnabel, Till Mittank‐Weidner, Jan‐Christoph Simon, Sonja Grunewald

**Affiliations:** ^1^ Clinic and Polyclinic for Dermatology Venereology and Allergology Leipzig University Hospital Leipzig Germany

**Keywords:** CO_2_ laser treatment, Rhinophyma, shave excision, skin surgery

## Abstract

**Background:**

Currently, there are no effective drug therapy options that lead to the regression of rhinophyma. Many surgical procedures for treating rhinophyma have been published; however, there are limited data on their effectiveness. In addition, these treatment options often lead to persistent hypopigmented scarring if excessive ablation or heat is applied.

**Patients and Methods:**

To prevent excessively deep ablation and enable precise nasal contouring, a combination therapy was developed, integrating classic rhinoshave for coarse ablation followed by laser fine contouring. Fractional ablation was performed to minimize heat accumulation. Here, we report on 45 patients with pronounced rhinophyma who underwent this treatment at the Dermatology Clinic of Leipzig University between 2016 and 2024. The median follow‐up period was 51 months (range: 6–96 months).

**Results:**

The majority of the patients were either very satisfied or satisfied with the postoperative outcome (n = 26; 92.3%). One case of postoperative hemorrhage was reported (2.2%). There was no wound infection observed, and the recurrence rate was 17.9%.

**Conclusions:**

The combination of rhinoshave and fractional ablative CO_2_ laser treatment is a feasible and safe therapeutic procedure and ensures high patient satisfaction with a low recurrence rate.

## INTRODUCTION

Rhinophyma is the term that is used to describe the manifestation of rosacea of the nose associated with diffuse connective tissue and sebaceous gland hyperplasia. The pathogenesis of this disorder has not yet been fully clarified.^1^ A multifactorial genesis is assumed to result in this condition, which consists of neurovascular dysregulation, a disorder of the innate and acquired immune system, an inflammatory reaction to cutaneous microorganisms and genetic factors. Rhinophyma particularly affects men (ratio of 5 : 1 to 30 : 1) over the age of 40 years with skin type I or II.^2^, ^3^, ^4^ Only the lower two thirds of the nose with involvement of the nostrils are affected.^5^ The *Rhinophyma Severity Index* (RHISI), which uses five clinical grades based on the degree of skin thickening, the presence of fissures and nodular proliferation has been used to describe the degree of rhinophyma. The maximum score for this index is six points. Additionally, an extra point may be awarded for the presence of marked asymmetry, multiple cysts, or larger vessels.^6^

Rhinophyma is often perceived as a considerable nuisance due to its central location in the midface region. In addition to impaired nasal breathing, the detection of unpleasant odors can also be a reason for surgical treatment. The RHISI score can be determined for objective assessment. For the RHISI score, values > 3 (which can represent incipient impairment of nasal breathing or significant cosmetic impairment) confirm the need for surgery.

Currently, there are no drug therapies that lead to a regression of a pronounced rhinophyma. Numerous physically destructive therapy procedures and combinations of these procedures have been described, which essentially utilize the following three different procedures: purely mechanical ablation, radiofrequency ablation or laser ablation.^7^

Mechanical ablation is classically performed using rhinoshave and dermabrasion.^8^ The disadvantage of this technique includes the limited possibility of fine contouring, particularly on the nostrils and at the nasal entrance, as well as the lack of hemostasis.

Alternatively, ablation can be performed using cryosurgery or the application of heat.^9^ Various types of scalpels are available, particularly those that can be heated to 150–200°C (Shaw scalpel, C2Dx, USA) or those that can use ultrasound (Harmonic ultrasound scalpel, Ethicon Endo‐Surgery, USA) to simultaneously achieve hemostasis.^10^, ^11^ Electrosurgical approaches use high‐frequency alternating current to simultaneously cut tissue and achieve haemostasis.^12^ However, the abovementioned heat‐based procedures cause considerable thermal damage that is sometimes difficult to control. This damage is the main cause of the frequently generated depigmented scars with respect to these treatments.

In laser treatment, the use of fractionated or non‐fractionated CO₂ lasers, particularly erbium:YAG lasers, has been described.^13^, ^14^, ^15^ The inability to perform histological tissue examinations and possible thermal damage to larger areas are disadvantages associated with this method.

Hydrosurgery systems (such as the VERSAJET™ system, Smith & Nephew, UK) are used less frequently; moreover, in addition to the investment costs, this system also has disadvantages regarding fine contouring.^16^ The use of cold plasma (such as J‐plasma, Apyx Medical Corporation, USA) has also been described.^17^ Furthermore, the use of X‐rays is now considered to be obsolete in view of the potential for cancer development, particularly in older individuals with lighter skin types.^18^

## SURGICAL TECHNIQUE

After careful color marking of the edges of the rhinophyma, approximately 30 mL of a tumescent local anesthetic solution (containing lidocaine, ropivacaine, suprarenin, and Ringer's solution) is infiltrated until distinct brightening and swelling of the nose are observed. After the administration of local anesthesia, excess tissue is removed via a hand dermatome or scalpel (blade no. 10) to achieve rough contouring of the nose. The deeper layers of the skin are carefully preserved to reduce scarring and to facilitate re‐epithelialization.

**FIGURE 1:**
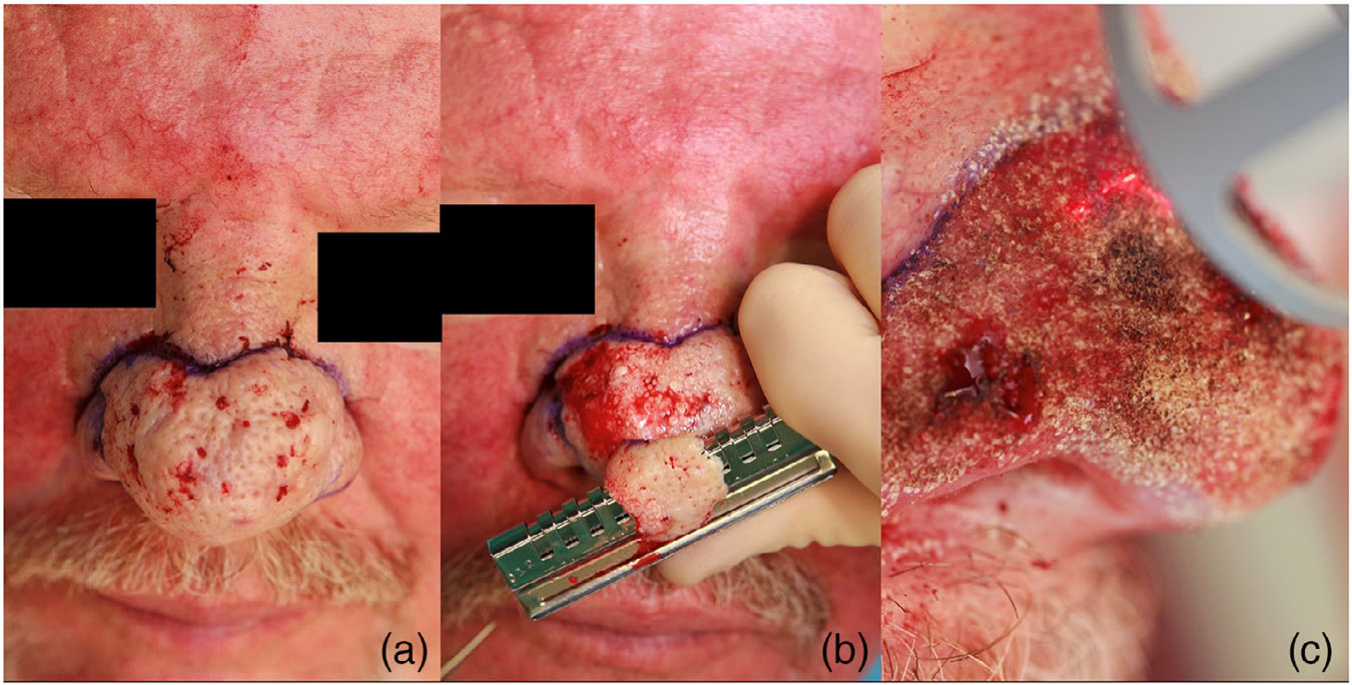
(a) The surgical area was marked after administration of local anesthesia. (b) Ablation was performed via a hand dermatome. (c) Fractional CO_2_ laser application for hemostasis and fine contouring. Fine contouring was performed according to anatomical conditions and personal photographs that were taken before the onset of the disease, with deeper ablations achieved using multiple passes of the laser.

These steps are immediately followed by fractional ablative CO_2_ laser therapy for fine contouring, which is particularly focused on the tip of the nose and the nostrils (PIXEL CO2, Alma Laser, 30 W, 1 ms, 5‐density; the spot size for the laser was chosen to vary between 4 and 6 mm and was observed to be even smaller if the sulcus alaris was restored (Figure 1). As CO_2_ lasers can determine different parameters (such as fluence/power) and are only comparable to a very limited extent, parameters with high power or energy density and relatively short pulse durations are generally suitable for this purpose, and the density of the ablation channels should be high. Multiple passes of the laser are necessary; specifically, a deeper contour that is obtained corresponds to a greater number of passes. This procedure is usually sufficient for hemostasis. Furthermore, bipolar coagulation is only selectively used on individual vessels when necessary.

In principle, laser therapy can also be performed in full ablation mode, which would represent an even faster process. However, this method would result in greater development of heat, thereby increasing the risks of permanent scars.

After the procedure, the wound is covered with paraffin‐coated gauze (Jelonet™, Smith & Nephew, UK) and antibacterial cream (Fucidine^®^, Leo Pharma, Denmark) for 1–2 days, followed by secondary healing, during which crusts remain in place, over a period of 1–2 weeks. All of the removed tissue is histologically examined to ensure that clinically occult epithelial neoplasia is not overlooked. Furthermore, the use of sun protection products is routinely recommended after wound healing is completed.

## PATIENTS AND METHODS

In addition to the retrospective analysis of the patient population, including imaging documentation, we conducted a standardized telephone follow‐up survey, in which verbal permission was given to participate in this data collection. We asked the patients to provide a graduated assessment of satisfaction (very satisfied, satisfied, neutral, dissatisfied and very dissatisfied). Yes‐no questions were used to evaluate the occurrence of aesthetically unpleasant scars, recurrence of the disease with the possible need for further surgical treatment, the occurrence of complications, the feasibility of surgery under local anesthetic administration, the likelihood of recommendation of the treatment and a self‐assessment of the duration of wound treatment. A corresponding vote by the ethics committee of the University of Leipzig with respect to the data collection and evaluation was available (268/24‐ek). Additionally, the surgical technique remained unchanged throughout the entire study period. There was no change in the use of instrumentation, and all of the rhinophyma procedures were performed by the same surgeon during this period.

## RESULTS

In this monocentric retrospective survey, 46 patients with pronounced rhinophyma (RHISI score > 3) who underwent surgical treatment with this technique at the Leipzig University Dermatological Clinic between August 2016 and February 2024 were evaluated. Due to the severity of the condition, these treatments were primarily applied to inpatient cases. At the time of the short‐term follow‐ups conducted a few months after treatment, all patients achieved highly satisfactory results.

A telephone survey was conducted to assess the long‐term postoperative results. Ultimately, 27 out of 45 patients (60%) were successfully contacted. The median follow‐up period was 51 months (range: 6–96 months). One patient died during the follow‐up from a cause unrelated to treatment. The data collected were analyzed using SPSS 29 (IBM, USA).

**FIGURE 2:**
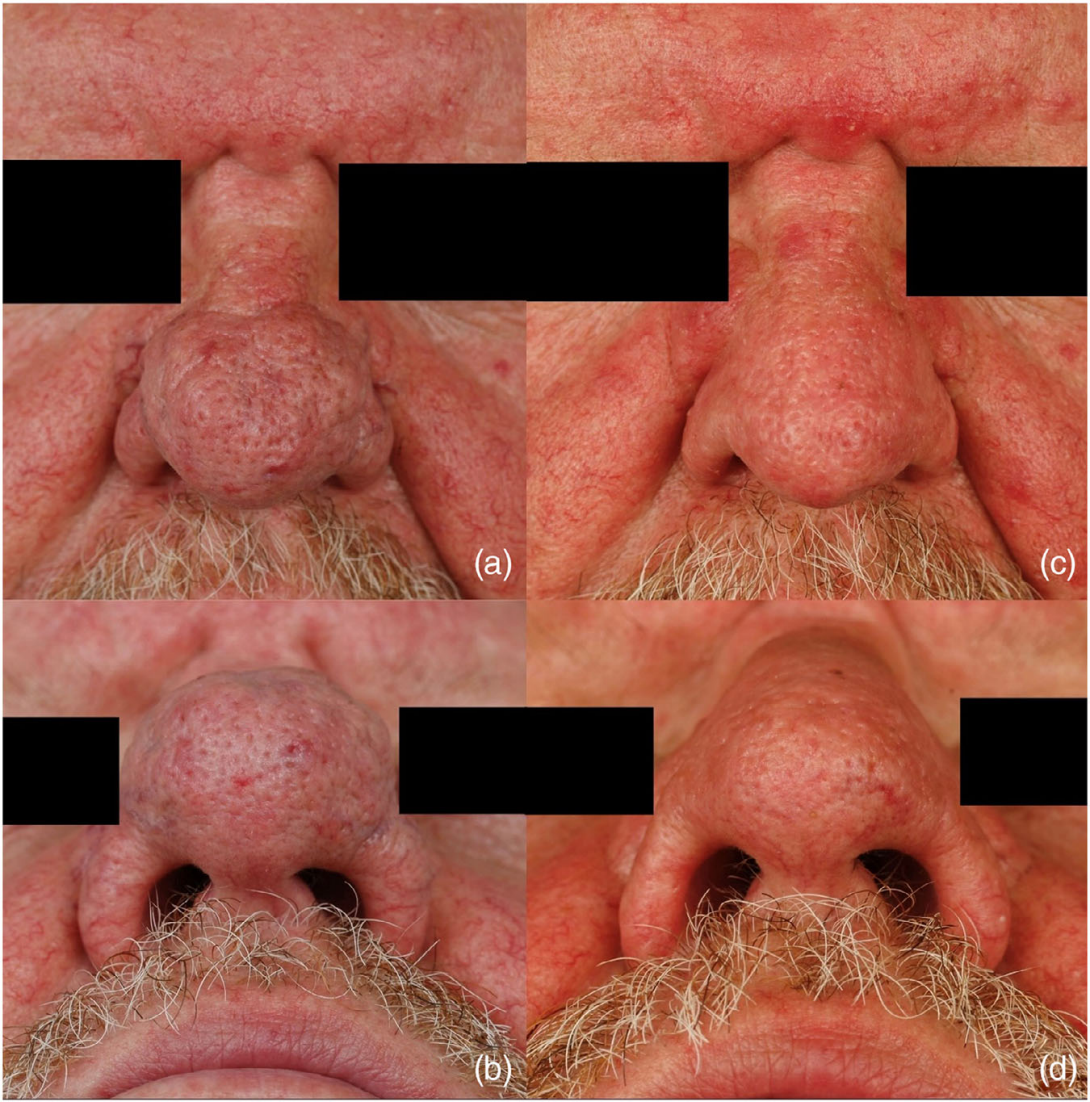
(a) Patient example 1: frontal preoperative image. (b) Nasal entrance preoperative image. (c) Frontal result at 3 months postoperative. (d) Nasal entrance result at 3 months postoperative.

**FIGURE 3:**
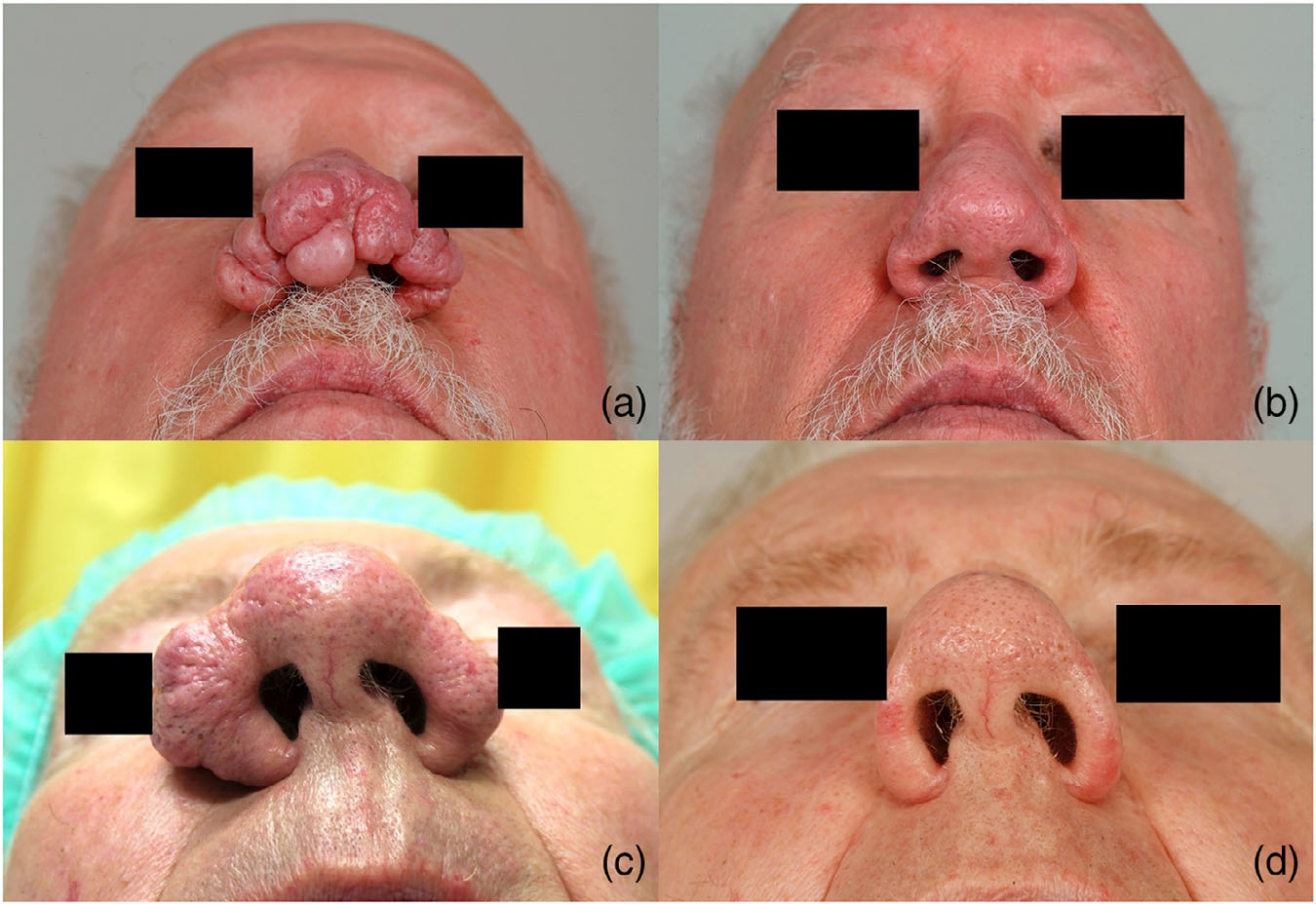
(a) Patient example 2: preoperative image. (b) Results at 3 months postoperative. (c) Patient example 3: preoperative image. (d) Results at 3 months postoperative.

The majority of our patients were male (n = 43; 91.5%) and had advanced rhinophyma (mean RHISI score: 4.4) (Table 1). The median age at the time of surgery was 67 years (37–81 years). The majority of patients were either “very satisfied” or “satisfied” with the long‐term postoperative outcome (n = 25; 92.6%). No patient suffered from functional impairments. Two patients (n = 2; 7.4%) were “dissatisfied” with the postoperative cosmetic results, both of them experienced rhinophyma recurrence. One of the “dissatisfied” patients reported that the procedure under local anesthesia was stressful, while the other patient mentioned aesthetically disturbing scarring. No patient was “very dissatisfied” (Table 2). A moderate postoperative hemorrhage occurred in one patient under the antiplatelet agent clopidogrel, which required bipolar hemostasis. There was no postoperative infection observed in any of the patients (Table 3). The majority of patients did not find the operation under local anesthesia to be stressful (n = 24; 88.9%) and would recommend the surgical procedure to others (n = 25; 92.6%). The wound healing time was prolonged in only one patient. Moreover, the recurrence rate was 17.9%. One patient reported subjectively disturbing scarring (n = 1; 3.7%) without a desire for follow‐up treatment (Figures 2, 3).

**TABLE 1:** Epidemiological data of 46 patients with rhinophyma.

Gender	
Male	43 patients (95.6%)
Female	2 patients (4.4%)
Mean RHISI score	4.4
Average age at the time of surgery	63.7 years
Median age at surgery	67 years

**TABLE 2:** Patient satisfaction.

Satisfaction level	n (%)
Very satisfied	19 (70.4%)
Satisfied	6 (22.2%)
Neutral	0 (0%)
Dissatisfied	2 (7.4%)Reasons for dissatisfaction (multiple choice possible): ‐ Recurrence: 100% ‐ Scarring: 50% ‐ Stress due to local anesthesia: 50%
Very dissatisfied	0 (0%)
Aesthetically disturbing postoperative scarring	1 (3.7%)
Occurrence of a recurrence	5 (17.9%)
Did you find the operation under local anesthetic stressful?	Yes: 3 (11.1%)
	No: 24 (88.9%)
Did you find the wound healing time to be long?	Yes: 1 (3.7%)
	No: 26 (96.3%)
Would you recommend the surgical procedure to others?	Yes: 25 (92.6%)
	No: 2 (7.4%)

**TABLE 3:** Postoperative complications.

Complication	n (%)
Postoperative bleeding	1 (3.5%)
Infection	0 (0%)

## DISCUSSION

Very few longer‐term postoperative results after rhinophyma treatment have been previously published. The recurrence rate of our combined method is significantly lower compared to shave excision alone (17.9% vs. 47%, respectively).^6^ The additional laser treatment appears to provide clinical value for this disorder. It is possible that the thermal effect of the laser (particularly in deeper skin layers) achieves long‐term stabilization of the collagen fiber scaffold, which has a preventative effect on the development of rhinophyma. Long‐term recurrence rates for other methods (particularly cold plasma) have not been reported in the literature.

We were unable to identify any typical long‐term side effects of other rhinophyma treatment methods, particularly with respect to hypopigmentation. Only one case reported persistent scarring, in which the rhinoshave was accidentally removed at too great a depth.

The limitations of the present study include its retrospective nature and the lack of direct comparisons with other surgical methods.

Compared with the use of CO_2_ laser therapy alone in fully ablative mode (continuous cutting mode and resurfacing mode), the results of the reported method are comparable. In a study that included 124 patients, good to very good results were reported in 118/124 patients (95.2%), and poor results were reported in only six patients (4.8%) at 3 months after the last laser treatment, as assessed by the practitioners themselves. However, it should be noted that the treatment was performed more than once in nine patients (7.3%). Scarring and hypopigmentation were reported in four patients each (3.2%).^19^ In addition to the increased development of heat, the disadvantage of laser treatment alone is unavailability of histological processing; moreover, particularly in the case of larger exophytic rhinophyma, a longer processing time is needed.

With the combined method that is presented in this study, larger amounts of tissue can be gently and quickly removed; moreover, fine contouring along the convex and concave areas of the nostrils can be achieved using CO_2_ laser therapy, which is not possible with the use of shave excision alone.

The large number of published potential therapy options also suggests that there is not yet a single correct option for treatment. The combination of different methods makes it possible to utilize the advantages of each individual method.^20^, ^21^ The low proportion of female patients included in our cohort is consistent with reports from the literature.^7^

The use of tumescent local anesthesia allows for the simple performance of shave excision and laser treatment, with a reduced risk of bleeding that results from the increased secretion of adrenaline and long‐lasting anesthesia effects through the combined use of ropivacaine and lidocaine. The majority of our patients did not perceive this factor as being a burden.

Some surgeons prefer to perform the procedure under general anesthesia (possibly in addition to the use of local anesthesia), which is particularly observed in cases of very soft rhinophyma noses, with the goal of achieving better recognition of the actual shape of the nose.^22^

Furthermore, the original shape of the nose is difficult to recognize in the case of pronounced rhinophyma, even without the use of local tumescent anesthesia; additionally, old photos are usually more helpful in resolving this issue. Compared to general anesthesia, the cost and time savings of this procedure should be mentioned, with minimal risks of side effects. Tumescent local anesthesia is a safe and effective procedure, especially for older patients.^23^ The use of a long‐acting local anesthetic reduces the postoperative use of analgesics and the risk of adverse drug reactions.^23^

The combination of rhinoshave and fractional ablative fine contouring enables the gentle removal of even large amounts of tissue in advanced rhinophyma. Both histological examination and good hemostasis are achieved as a result of this procedure. In addition, fine contouring leads to very good cosmetic results with a minimized risk of scarring.

In our opinion, the method presented in this paper is particularly suitable for treating larger exophytic rhinophyma involving the nostrils.

To validate these study results, prospective randomized, multicenter studies (in particular, comparative studies of different treatment methods) would be helpful in the future.

## CONFLICT OF INTEREST STATEMENT

None.
